# Serum High-Sensitivity C-Reactive Protein Is Associated with Postoperative Psychiatric Status in Patients with Empty Nose Syndrome

**DOI:** 10.3390/diagnostics11122388

**Published:** 2021-12-18

**Authors:** Chia-Hsiang Fu, Hung-Chin Chen, Chi-Che Huang, Po-Hung Chang, Ta-Jen Lee

**Affiliations:** 1 Department of Otolaryngology—Head and Neck Surgery, Linkou Chang Gung Memorial Hospital, Taoyuan 333604, Taiwan; fufamily@cgmh.org.tw (C.-H.F.); b9602052@cgmh.org.tw (H.-C.C.); hcc3110@cgmh.org.tw (C.-C.H.); bc1766@gmail.com (P.-H.C.); 2 Graduate Institute of Clinical Medical Sciences, College of Medicine, Chang Gung University, Taoyuan 333604, Taiwan; 3 Department of Otolaryngology—Head and Neck Surgery, Xiamen Chang Gung Memorial Hospital, Xiamen 361000, China

**Keywords:** high-sensitivity C-reactive protein (hs-CRP), empty nose syndrome, biomarker, psychiatric, depression, Beck depression inventory-II (BDI-II), anxiety, Beck Anxiety Inventory (BAI), endonasal submucosal implantation, surgery

## Abstract

Many patients diagnosed with empty nose syndrome (ENS) later develop mental illness. The literature addressing biomarkers associated with postoperative psychiatric status is limited. This study aimed to assess the association between high-sensitivity C-reactive protein (hs-CRP) and psychiatric status after surgery in ENS. We recruited patients with ENS undergoing endonasal submucosal implantation. Their pre- and postoperative psychiatric status was evaluated using the Beck depression inventory-II (BDI-II) and the Beck Anxiety Inventory (BAI). Serum hs-CRP was analyzed one day before and one year after surgery. Of the 43 patients enrolled, all subjective measurements had improved (symptom scores decreased) significantly by the third month postoperatively and remained plateaued till 12 months. Those with preoperative hs-CRP levels > 2.02 mg/L were likely to remain depressive 1 year postoperatively. The regression model showed that a preoperative hs-CRP level > 2.02 mg/L was significantly correlated with postoperative depression in patients with ENS (odds ratio, 19.9). Hs-CRP level seems to be a feasible predictor of surgical outcome regarding improved depression in patients with ENS. Patients with higher preoperative hs-CRP levels should be monitored closely after surgery.

## 1. Introduction

Empty nose syndrome (ENS) is characterized by a subjective sensation of nasal blockage and suffocation and, paradoxically, a patent nasal cavity [1]. ENS was first described in 1994, and the term was used to describe the wide nasal passages observed on endoscopy or imaging [2]. In addition to nasal and pharyngeal symptoms, such as dyspnea, air hunger, dryness, and hyposmia, ENS also causes serious psychological and emotional disorders, such as chronic fatigue, depression, anxiety, and frustration [3]. However, the diagnosis of ENS by physical examination alone is unreliable. The objective findings correlate poorly with the subjective symptoms of patients with ENS [4]. Thus, the improvement of the subjective symptoms became the primary measurement of treatment outcome.

Mental illness has been observed to develop in about 70% of patients with ENS [5]. These patients experience a heavy psychiatric burden, which reduces their productivity at work markedly and affects their activities of daily living [6]. Lemogne et al. presented a patient with ENS with psychosomatic disorders who benefited from cognitive behavior therapy and complementary antidepressant treatment [7]. Bastier et al. reported that the impact scale domain, which reflects emotional disturbance and functional impairment, showed significant improvement after the submucosal implantation of β-tricalcium phosphate grafts in patients with ENS [8]. A previous study showed that depression and anxiety estimated by the BDI-II and BAI could be alleviated by surgery without additional antipsychotic medication [5]. 

Several studies have demonstrated the effectiveness of inferior meatus augmentation through surgical submucosal implantation as a treatment for ENS [9,10,11]. Surgical intervention involves an implant secured within a submucosal pocket to re-build the geographic contour of the nasal cavities, resulting in increased nasal resistance and effective redirecting of the inspired air via laminar flow into the nasopharynx [12]. The major purpose of surgical treatment is to reconstruct a proper nasal airway, to increase resistance and deflect airflow from insensitive tissue to unoperated areas [13]. This procedure seems effective and safe, leading to significant improvements in subjective symptoms, quality of life, and the severity of depression and anxiety [5,13]. Patients with positive cotton tests were likely to benefit from surgical treatment [5]. However, to date, there is limited literature addressing the prediction of surgical outcomes related to nasal symptoms and psychiatric status in patients with ENS. Our previous investigation showed that ENS patients with submucosal lateral nasal wall implants had higher improvements in rhinological symptoms and sleep function compared to patients received nasal floor implants [10]. The presentation of a thermoreceptor, transient receptor potential channel melastatin 8 (TRPM8), decreased in the remnant nasal tissue, might have some role in psychiatric symptom scores for ENS patients [14], but is not practical to apply in a preoperative consultation of surgical prognosis. A feasible, reliable biomarker is crucial for development of diagnosis and prediction of treatment response.

Several biomarkers associated with depression and anxiety have been proposed. Neurotransmitters such as serotonin IA receptor [15] and some neurotrophic factors including brain-derived neurotrophic factor (BDNF), nerve growth factor (NGF), glial cell line-derived neurotrophic factor (GDNF), and vascular endothelial growth factor (VEGF) [16,17,18,19] have been investigated as potential biomarkers in psychiatric diseases. Changes in DNA, epigenetic modifications (such as DNA methylation), and microRNA (miRNA) level also have been used as biomarkers in psychiatric disorders [20,21,22,23]. Nevertheless, the sensitivities and specificities of these novel biomarkers for psychiatric disorders still need larger sample size to drive more definite conclusions [24]. On the other hand, our histology analysis showed that over 80% ENS patients presented abundant chronic lymphocytic infiltration in their nasal mucosa, indicating a chronic inflammation status in the development of ENS [14]. Inflammatory cytokines, including interleukins (ILs) and C-reactive protein (CRP) that have been applied in our daily practice have been proposed to be related to depression and anxiety disorders [25,26,27]. 

CRP is a systemic biomarker for inflammation, and serum levels increase in response to tissue damage and stress. Accumulating evidence shows that inflammation plays a role in the chronic course of depression [28]. Inflammatory response activation might result in treatment-refractory emotional disorders via neurodegenerative and oxidative stress pathways [29]. Elevated CRP levels have been found in some patients with depression without autoimmune, infectious, and malignant diseases [30]. Depression was associated with a higher CRP level in a cross-sectional study of 14,276 patients in the United States [31]. An increased CRP level has been documented to be a useful biomarker for predicting a nine-year major depressive disorder diagnostic status change [32]. Pasco et al. considered high-sensitivity C-reactive protein (hs-CRP) as an independent risk marker for de novo major depressive disorder in women after 5827 person-years of follow-up [33]. Further, some patients with ENS also experienced psychiatric disorders [5,6]. A connection between low-grade inflammation and mental illness has been proposed, and CRP is a well-established biomarker of low-grade inflammation [34,35]. The central nervous system is an immune-privileged site that may become the source of inflammatory factors to maintain the immune tolerance. After a period of tolerance, inflammatory factors increase eventually, leading to inflammatory exacerbation and contributing to a wide range of psychiatric diseases. Inflammatory pathways and immune systems are dysregulated in some psychiatric illnesses such as anxiety and stress [36]. Inflammatory biomarkers could be measured peripherally (saliva, serum) or centrally (cerebrospinal fluid or brain tissue), and serum biomarker measurement would be more practical. Therefore, this study aimed to determine the association between serum hs-CRP levels and psychiatric status, such as depression and anxiety, before and after surgical intervention in patients with ENS. 

## 2. Materials and Methods

### 2.1. Patient Enrollment

This was a prospective study approved by the Chang Gung Memorial Hospital Institutional Review Board (IRB No. 201802147A3). Patients with a history of turbinectomy and typical ENS symptoms, such as paradoxical nasal obstruction, air hunger, dryness, headache, or emotional disorders, were diagnosed with ENS. We enrolled those who had positive cotton tests and Empty Nose Syndrome 6-Item Questionnaire (ENS6Q) scores of at least 11, who eventually underwent endonasal submucosal implantation [37]. The exclusion criteria were (1) a history of any psychiatric disorders or antipsychotic medication use before the first turbinectomy surgery; (2) congenital craniofacial anomaly or nasal deformity resulting from trauma or rhinoplasty; (3) concomitant sino-nasal diseases, such as nasal polyps or rhinosinusitis; and (4) a history of sino-nasal neoplasm or radiation therapy.

### 2.2. Associated Parameters

The patients’ age, gender, and smoking status were recorded. Rhinomanometry and venous blood collection were performed 1 day before surgery. Besides routine blood tests, allergy tests and hs-CRP levels were also analyzed. Reliable high-sensitivity assays for CRP are now widely accessible with the particle-enhanced immunonephelometric assay. The sensitivity has improved from 6 to 10 to 0.15 to 0.18 mg/L [38]. Peripheral blood was drawn and then centrifuged at 3000 rpm at 4 °C for 15 min, and aliquots were stored at −70 °C. In this study, the high-sensitivity immunonephelometric assays for CRP (Nanopia CRP; Sekisui Medical Co., Tokyo, Japan) were performed by automated analyzers with the lowest detection limit of 0.2 mg/L. Postoperative serum hs-CRP levels were collected when patients had fulfilled the 12-month follow-up period.

### 2.3. Measurements of Nasal–Facial Symptoms and Psychiatric Status

Subjective symptoms were measured using the Sino-Nasal Outcome Test-25 (SNOT-25) as well as the validated ENS6Q. SNOT-25 was modified from SNOT-20 by Houser and consists of 25 questions, scoring from 0 (no symptoms) to 5 (severe symptoms), to gauge the five most frequent symptoms related to ENS: dryness, difficult nasal breathing, suffocation, open nose, and nasal crusting [4]. ENS6Q contains six items (dryness, sense of diminished nasal airflow, suffocation, nose feels too open, nasal crusting, and nasal burning) with a grading scale of 0–5 [37]. An ENS6Q score of 10.5 discriminated between patients with and without ENS, with a sensitivity and specificity of 86.7% and 96.6%, respectively [37].

The patients’ psychiatric status was evaluated using the Beck depression inventory-II (BDI-II) and Beck Anxiety Inventory (BAI). The BDI was one of the most widely used self-reporting scales to assess depression severity. It was first developed by Beck et al. in 1961 and was revised as the BDI-II in 1996. It includes 21 items rated on a 4-point scale ranging from 0 to 3 based on severity. Final scores of 0–13, 14–19, 20–28, and 29–63 indicate normal, mild, moderate, and severe depression, respectively [39]. Similarly, the BAI was developed by Beck et al. in 1990 and has been an indicator of anxiety in patients with different anxiety disorders. There are 21 items rated on a scale from 0 to 3. Total scores of 0–7, 8–15, 16–25, and 26–63 indicate normal, mild, moderate, and severe anxiety, respectively [40]. All patients completed the SNOT-25, BDI-II, and BAI 1 week preoperatively and repeated all surveys (including ENS6Q) at 3, 6, and 12 months postoperatively. There is no recommended follow-up period in the literature for patients with ENS undergoing surgery. Our previous study showed that rhinological and psychiatric symptoms of ENS patients reached a plateau by the third month after surgical treatment and remained there [41]. Therefore, a follow-up period of 12 months postoperatively was set as the primary endpoint of this investigation.

### 2.4. Surgical Intervention and Postoperative Protocol

All patients underwent endonasal submucosal implantation with porous high-density polyethylene (Ultra-Thin Sheet Medpor^®^, Porex Surgical, Inc., Newnan, GA, USA) by the same physician. We have described the surgical procedure in previous studies [6,9]. An incision line is made on the lateral nasal wall over the pyriform aperture with a miniature blade (Surgistar^TM^, Inc., Knoxville, TN, USA) with endoscopic assistance. The line is located on the inferior wall of the inferior meatus as close to the lateral wall as technically possible and then extended laterally against the lateral nasal wall. A submucosal pocket is created with a freer suction elevator. The implants are trimmed into small pieces, between 8 × 25 mm^2^ and 8 × 40 mm^2^, and used to fill the submucosal space. The volume of Medpor^®^ grafts used to form an ideal implant contour is based on the length of the patient’s inferior meatus. Finally, the elevated mucosal flap is repositioned to make the augmentation surface intact. We stabilize the implants by placing nasal packing against the implant in the lateral wall. 

All patients were discharged on the same day from the post-anesthesia care unit. Patients received postoperative nasal debridement weekly in the first month. Regular endoscopic follow-up was carried out monthly for 6 months and every 2 months after that.

### 2.5. Statistical Analysis

The Wilcoxon signed-rank test was used to compare the pre- and postoperative subjective assessments and hs-CRP levels. Data were presented as mean ± standard deviation (SD). The association between hs-CRP and other potential medical factors was analyzed using the Spearman correlation test and the Mann-Whitney U test for continuous and categorical variables, respectively. Multivariate logistic regression was performed to identify independent patient characteristics associated with postoperative psychiatric status. The preliminary model included a binary measurement of either remaining depressed or in an anxiety state postoperatively as the main dependent variable of interest. All independent cofactors were screened for univariate significance (*p* < 0.05). The final logistic regression model was created using forward stepwise selection (*p* = 0.05) to identify independent patient characteristics associated with postoperative depression. The odds ratio (OR) and 95% confidence interval (CI) were also reported. 

Moreover, using the receiver operating characteristic (ROC) curve, the ideal cut-off value of the hs-CRP level for predicting the postoperative psychiatric status was determined based on Youden’s index. Two-tailed *p* < 0.05 was considered statistically significant. Statistical analyses were performed using the SPSS 16.0 statistical package for Windows (SPSS Inc., Chicago, IL, USA).

## 3. Results

### 3.1. Patient Characteristics

**Figure 1:**
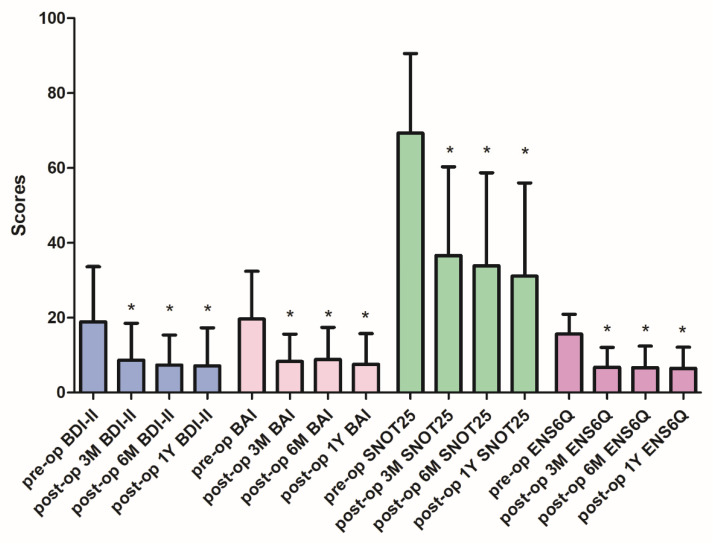
The subjective measurements before and after surgical treatment. The postoperative BDI-II, BAI, ENS6Q and SNOT-25 scores significantly improved at the third month and remained plateaued until one year after surgery. Pre-op: preoperative; post-op: postoperative; m: month; BDI-II: Beck depression inventory-II; BAI: Beck anxiety inventory; SNOT-25: Sino-Nasal Outcome Test-25; ENS6Q: Empty Nose Syndrome 6-Item Questionnaire. Data were presented as mean ± standard deviation (SD). * *p* < 0.05 compared with preoperative status.

Forty-nine patients who met the inclusion criteria over 2 years and received endonasal submucosal implantation treatment were enrolled. Two patients were lost to postoperative follow-up, and four who refused a postoperative serum test were excluded. Eventually, 43 patients (37 men and six women) were included in the statistical analysis. Table 1 shows the patients’ demographic data, laboratory parameters, and preoperative rhinology and psychiatric assessments. The mean age was 44.7 (range, 22–68) years, and the preoperative serum hs-CRP level was 1.41 (range, 0.20–4.06) mg/L. Before surgical treatment, only 41.9% and 18.6% of these ENS patients were at non-depression and non-anxiety status, respectively. The postoperative ENS6Q and SNOT-25 scores markedly improved at the third month (both *p* < 0.001) and remained plateaued until one year after surgery (Figure 1). Similarly, both the BDI-II and BAI scores improved significantly and plateaued by the third month (both *p* < 0.001; Figure 1). No significant differences were found in the ENS6Q, SNOT-25, BDI-II, and BAI scores between 3, 6, and 12 months following surgery. Similar results were observed in our previous study [41].

### 3.2. The Change in Serum hs-CRP Level after Surgery

**Figure 2:**
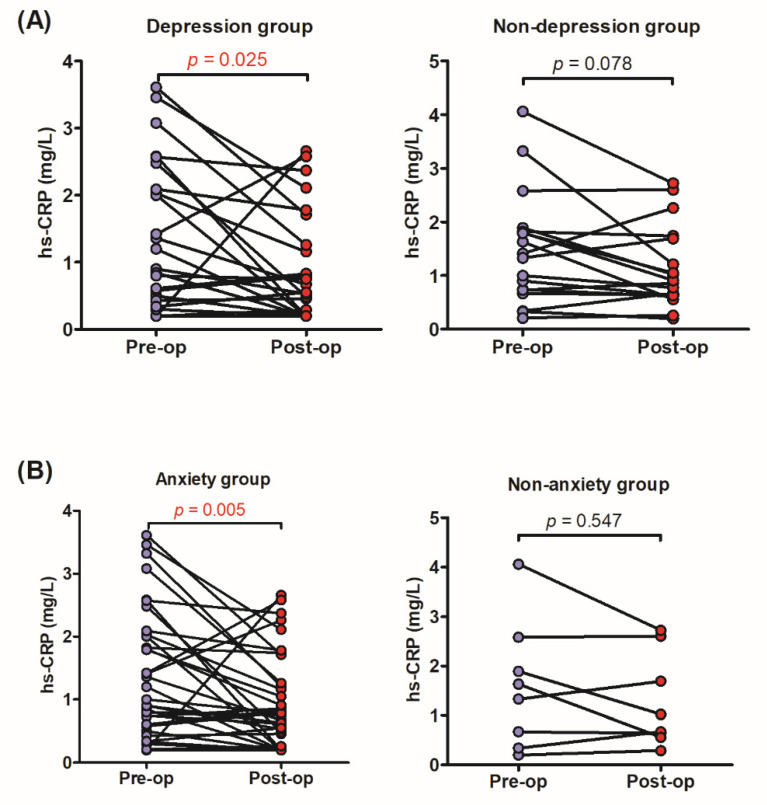
The change of serum hs-CRP level in preoperative (**A**) depression group (left panel), non-depression group (right panel), (**B**) anxiety group (left panel), and non-anxiety group (right panel). The postoperative serum hs-CRP significantly decreased in the depression and anxiety groups but not in the non-depression and non-anxiety groups. hs-CRP: high-sensitivity C-reactive protein; pre-op: preoperative; post-op: postoperative.

Among the 43 patients who had the preoperative and one year postoperative follow-up measurement of serum hs-CRP, 28 (65.1%) had a decreased level after surgical treatment. The postoperative serum hs-CRP decrease was statistically significant (*p* = 0.006). The trend of postoperative serum hs-CRP change according to preoperative psychiatric status was further investigated. The postoperative serum hs-CRP significantly decreased in the depression group but not in the non-depression group (*p* = 0.025 and 0.078, respectively; Figure 2A). Similarly, if we categorized the patients based on preoperative anxiety status, the postoperative serum hs-CRP markedly decreased in the anxiety group but not in the non-anxiety group (*p* = 0.005 and 0.547, respectively; Figure 2B).

### 3.3. Correlation and ROC Curve Analysis for Preoperative hs-CRP

**Figure 3:**
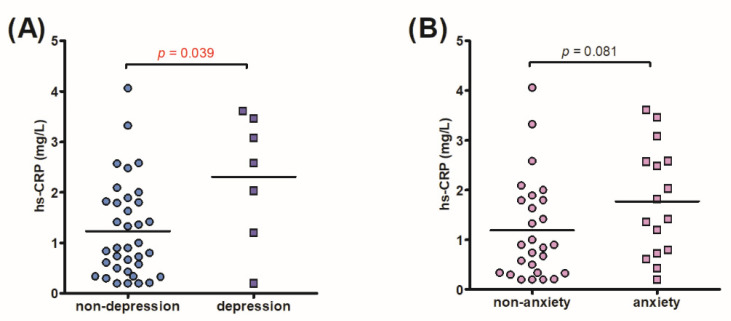
The differences in the preoperative serum hs-CRP level between postoperative (**A**) non-depression group and depression group, and (**B**) non-anxiety group and anxiety group. Compared to the postoperative non-depression group, patients had significantly higher preoperative serum hs-CRP level in the postoperative depression group. As to the serum hs-CRP levels between the non-anxiety and anxiety groups, the difference did not reach statistical significance. hs-CRP: high-sensitivity C-reactive protein.

**Figure 4:**
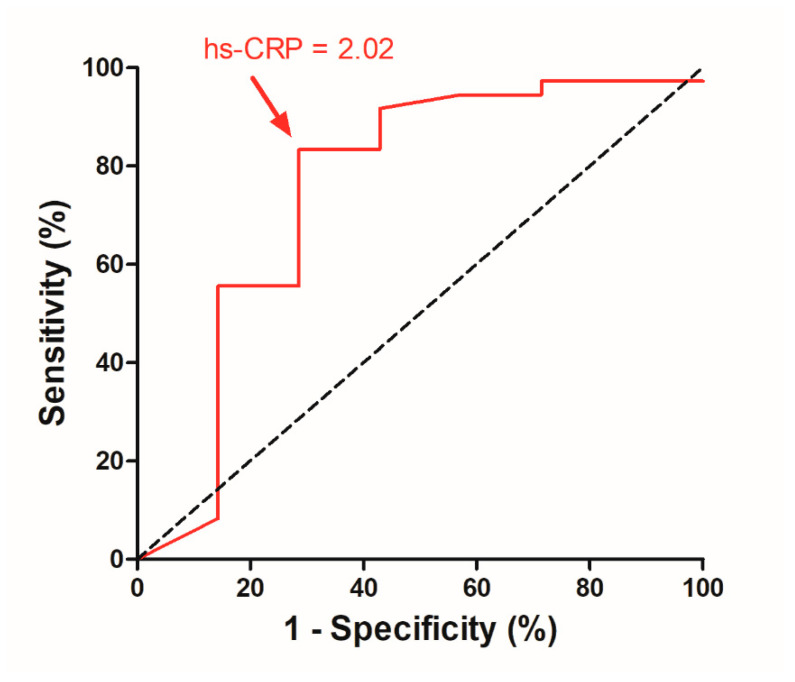
Receiver operating characteristic curve analysis for preoperative hs-CRP in predicting postoperative depressive status. A cutoff value of preoperative hs-CRP value at 2.02 mg/L was obtained to have optimal sensitivity and specificity. hs-CRP: high-sensitivity C-reactive protein.

Table 2 shows the relationship between the preoperative hs-CRP level and potentially associated medical factors. Preoperative hs-CRP levels showed no significant correlation with demographic data and the preoperative ENS6Q, SNOT-25, BDI-II, and BAI scores (*p* = 0.704, 0.099, 0.571 and 0.755, respectively). After surgical treatment, 7 and 16 patients remained in depression and anxiety, respectively, one year later. The only factor that significantly correlated to serum hs-CRP was postoperative depression status (*p* = 0.039). A higher preoperative hs-CRP level was associated with postoperative depression (*p* = 0.039; Figure 3A). The correlation between the hs-CRP level and postoperative anxiety reached a near significance (*p* = 0.081; Figure 3B). These results indicate that a higher preoperative hs-CRP may have a role in postoperative psychiatric status. By contrast, the postoperative ENS6Q and SNOT-25 scores were not associated with the hs-CRP level (*p* = 0.830 and 0.467, respectively). ROC curve analysis showed an association between postoperative depression 1 year postoperatively and the preoperative hs-CRP cut-off level of 2.02 mg/L (*p* = 0.038; Figure 4). This indicated that patients with ENS were more likely to return to a non-depressive status after 1 year when they had a preoperative hs-CRP value < 2.02 mg/L, with 83.3% sensitivity and 71.4% specificity (Table 3). The positive predictive value (PPV) and negative predictive value (NPV) for applying the preoperative hs-CRP level at 2.02 mg/L to predict a non-depressive postoperative status of patients with ENS would be 93.8% and 45.4%, respectively.

### 3.4. Regression Analysis for Postoperative Depression

Univariate logistic regression analysis showed postoperative depression at 1 year was associated with age, allergy status, a higher preoperative hs-CRP level (>2.02 mg/L), and preoperative subjective measurements (ENS6Q, SNOT-25, BDI-II, and BAI scores) (all *p* < 0.001). The multivariate logistic regression model further showed that age and a preoperative hs-CRP level > 2.02 mg/L were significantly correlated with postoperative depression status (*p* = 0.044 and 0.008, respectively) (Table 4). The OR of a higher preoperative hs-CRP level was 19.9 (95% CI, 2.2–182.7), resulting in an approximately 20-fold chance of remaining in postoperative depressive status for patients with ENS with a preoperative hs-CRP level > 2.02 mg/L.

## 4. Discussion

To our knowledge, this is the first study addressing serum biomarkers for the surgical prognosis of ENS. CRP is a systemic marker of inflammation, and it increases in the peripheral blood following infection, trauma, and/or tissue damage [42]. In the past, no adequate laboratory assay methods could detect CRP serum levels within 10 mg/L. However, the progress of high-sensitivity assays has made the detection of mild elevations of CRP < 10 mg/L possible [43]. The high-sensitivity immunonephelometric assays applied for CRP in this study could reach a lowest detection limit of 0.2 mg/L. The present study showed that the preoperative hs-CRP level was associated with postoperative psychiatric outcomes in patients with ENS. Patients with a preoperative hs-CRP level less than 2.02 mg/L had an encouraging probability of 93.8% of regaining non-depressive status after surgical treatment. The proportion decreased to 54.6% (6 of 11) to acquire a normal psychiatric status if their hs-CRP was > 2.02 mg/L preoperatively. Therefore, the preoperative hs-CRP level seems to be a feasible predictor for postoperative psychiatric outcome and may be valuable in screening suitable surgical candidates in patients with ENS. More definite results might be expected if a more precise assay for CRP detection in trace amount available.

To date, the pathophysiology of psychiatric illness in patients with ENS has not been well clarified. The persistent sensation of poor breathing, air hunger, and suffocation preoccupies patients with ENS [1]. This progressively added to their frustration and feeling of hopelessness. In this study, before surgical treatment, 58.1% and 81.4% of patients met the criteria for depression and anxiety, respectively. Similar to previous studies, a significant proportion of ENS patients experienced heavy psychiatric burdens [5,6]. Freund et al. observed the activation of the amygdala and limbic system during resting respiration in patients with ENS with functional magnetic resonance imaging [44]. The amygdala is responsible for the perception of emotions, such as anger, fear, and sadness. The amygdalar projections to the prefrontal and sensory cortices affect cognitive processes, such as attention and decision making, and hence plays an important role in social behavior and productivity [45,46]. The stimulation of the limbic system might explain why patients with ENS develop psychiatric illness and social withdrawal. 

It has been suggested that increased plasma CRP concentrations may interfere with functional connectivity between the amygdala and ventromedial prefrontal cortex, which correlates with psychiatric symptoms [47]. This may drive ENS patients suffering from both functional and psychiatric burdens. Consequently, this study assumed that certain pro-inflammatory pathways that promote increased CRP levels would disturb the integrity of the amygdala–prefrontal cortex circuit and add to the persistence of depression. Similarly, in patients with ENS in the present study, those with a higher preoperative hs-CRP level tended to remain depressive after surgical treatment. CRP elevation was documented to correlate significantly with depressive symptoms in a review article [48]. However, the sequential order between depression status and elevated CRP remains unclear and requires further research. Moreover, the correlation of serum CRP levels and the inflammatory status of nasal tissue in histologic analysis at different timepoints deserves further investigation for ENS patients.

In this study, the hs-CRP level decreased significantly one year after surgical treatment for ENS patients with preoperative depression or anxiety. Compared to those in preoperative non-depression and non-anxiety groups, serum hs-CRP levels in ENS patients with preoperative depression or anxiety were higher and therefore had more space to decrease after surgery. Further, baseline CRP levels have been reported to predict distinct treatment outcomes and influence antidepressant selection in patients with depression [49]. CRP levels were significantly elevated in treatment-resistant patients with depression but not so significantly in the treatment-responsive patients, compared with healthy individuals [50]. The present investigation showed similar results in that there was a 20-fold increase in risk for patients with ENS with preoperative hs-CRP levels > 2.02 mg/L of unsatisfactory psychiatric outcomes after surgery. Nevertheless, ENS patients with preoperative hs-CRP levels > 2.02 mg/L still could expect a 55% chance to remain non-depression status after surgical treatment. With more promising results for ENS, we may unveil the mysteries concerning this complex airway disease in the near future.

This study had a relatively small sample size (43 patients) and limited statistical power. However, most ENS results from an improper procedure and needs time to develop (sometimes years), making it difficult to enroll many patients in a short period. Nevertheless, a larger sample size and a longer follow-up period may be required to draw a more definite conclusion. Furthermore, our study did not include a control group because sham surgery would be considered unethical. The placebo effect should be considered. Further studies are warranted to investigate the mechanism and clinical effects of hs-CRP in the treatment of ENS.

## 5. Conclusions

For patients with ENS, serum hs-CRP may be a feasible indicator for predicting the surgical outcome of postoperative depressive status. It may be valuable to physicians in screening patients who are more likely to benefit from surgical intervention in recovering from depression caused by ENS.

## Figures and Tables

**Table 1:** Demographic data, laboratory parameters, and pre- and postoperative subjective assessments in our study subjects.

Variables	*n* = 43
Age, years (SD)	44.7 (13.2)
Gender, female: male (%)	6:37 (14.0:86.0)
Allergy status (%)	25 (58.1)
Smoking (%)	11 (25.6)
Serum hs-CRP, mg/L (SD)	1.41 (1.07)
Preoperative ENS6Q, score (SD)	15.7 (5.2)
Preoperative SNOT-25, score (SD)	69.3 (21.2)
Preoperative BDI-II, score (SD)	18.9 (14.7)
(1) normal (0–13) (%)	18 (41.9)
(2) mild degree (14–19) (%)	7 (16.3)
(3) moderate degree (20–28) (%)	5 (11.6)
(4) severe degree (29–63) (%)	13 (30.2)
Preoperative BAI, score (SD)	19.7 (12.8)
(1) normal (0–7) (%)	8 (18.6)
(2) mild degree (8–15) (%)	10 (23.3)
(3) moderate degree (16–25) (%)	11 (25.6)
(4) severe degree (26–63) (%)	14 (32.6)

All values are reported as mean (standard deviation) or number (percentage), as indicated. hs-CRP: high-sensitivity C-reactive protein; ENS6Q: Empty Nose Syndrome 6-Item Questionnaire; SNOT-25: Sino-Nasal Outcome Test-25; BDI-II: Beck depression inventory-II; BAI: Beck anxiety inventory.

**Table 2:** Correlation between the preoperative hs-CRP level and other parameters.

Variables	*p*-Value ^1^
Demographic factor:	
Age	0.537
Gender	1.000
Allergy	0.460
Smoking	0.889
Pre-op status:	
ENS6Q	0.704
SNOT-25	0.099
Depression (BDI-II > 13)	0.571
Anxiety (BAI > 9)	0.755
Post-op 12-month status:	
ENS6Q	0.655
SNOT-25	0.710
Depression (BDI-II > 13)	0.039 *
Anxiety (BAI > 9)	0.081

^1^ Spearman correlation and Mann-Whiney U test were applied for continuous and categorical variables, respectively. * *p* < 0.05 statistically significant. hs-CRP: high-sensitivity C-reactive protein; pre-op: preoperative; post-op: postoperative; ENS6Q: Empty Nose Syndrome 6-Item Questionnaire; SNOT-25: Sino-Nasal Outcome Test-25; BDI-II: Beck depression inventory-II; BAI: Beck anxiety inventory.

**Table 3:** Preoperative serum hs-CRP level according to postoperative depressive status.

hs-CRP Level	Non-Depression (BDI ≤ 13, *n* = 36)	Depression (BDI > 13, *n* = 7)	
≤2.02 mg/L (*n* = 32)	30	2	PPV: 30/32 = 93.8%
>2.02 mg/L (*n* =11)	6	5	NPV: 5/11 = 45.4%
	Sensitivity: 30/36 = 83.3%	Specificity: 5/7 = 71.4%	

hs-CRP: high-sensitivity C-reactive protein; BDI-II: Beck depression inventory-II; PPV: positive predictive value; NPV: negative predictive value.

**Table 4:** Logistic regression analysis of postoperative depression status and other potentially associated factors.

	Univariate Analysis			Multivariate Analysis		
	Estimate	(95% CI)	*p*-Value ^1^	Estimate	(95% CI)	*p*-Value ^1^
Age	1.1	(1.0–1.1)	<0.001 *	0.9	(0.8–1.0)	0.044 *
Gender		-	0.978		-	-
Allergy	102.3	(11.7–893.4)	<0.001 *		-	0.487
Smoking	31.4	(6.0–164.0)	<0.001 *		-	0.092
hs-CRP > 2.02 mg/L	140.4	(22.6–873.4)	<0.001 *	19.9	(2.2–182.7)	0.008 *
Pre-op ENS6Q	1.2	(1.1–1.4)	<0.001 *		-	0.786
Pre-op SNOT-25	1.1	(1.0–1.1)	<0.001 *		-	0.284
Pre-op BDI-II	1.1	(1.1–1.2)	<0.001 *		-	0.285
Pre-op BAI	1.2	(1.1–1.2)	<0.001 *		-	0.164

^1^ * *p* < 0.05 statistically significant; CI, confidence interval; NI, not included; NS, not significant; hs-CRP: high-sensitivity C-reactive protein; pre-op: preoperative; Pre-op: preoperative; ENS6Q: Empty Nose Syndrome 6-Item Questionnaire; SNOT-25: Sino-Nasal Outcome Test-25; BDI-II: Beck depression inventory-II; BAI: Beck anxiety inventory.

## Data Availability

The datasets generated during this study are available from the corresponding author on reasonable request.
